# Supplementary material for: Accurate prediction of thermoresponsive phase behavior of disordered proteins
Source: Protein Sci. 2025 Sep 13;34(10):e70284. doi: 10.1002/pro.70284 (PMC12432431; doi:10.1002/pro.70284)
Supplement: Supplementary file 1 — Data S1 [file PRO-34-e70284-s001.pdf]

Supporting Information for:
Accurate prediction of thermoresponsive phase behavior of disordered proteins

Ananya Chakravarti [1,2] and Jerelle A. Joseph [1,2,*]

[1] *Department of Chemical and Biological Engineering, Princeton University, Princeton, NJ 08544, USA*

[2] *Omenn–Darling Bioengineering Institute, Princeton University, Princeton, NJ 08544, USA*

(Dated: August 13, 2025)

Contents

I. Revised Wang–Frenkel Potential 1

II. Model Optimization 1

III. Model Testing 2

IV. CLOUD-FIT: Cloud Point Computations 11

V. Fitness Function Parameters for Model 3 12

References 22

I. Revised Wang–Frenkel Potential

Two versions of revised Wang–Frenkel potential accommodate for repulsive interactions. When ε_{ij} for a pair of beads becomes negative, the interaction between the beads becomes increasingly attractive (approaching ∞) as they move closer together, which would be unphysical (Fig. S1). To address this discrepancy, we create two versions of a revised potential that expresses repulsive interactions for bead pairs with negative ε_{ij} (Eqs. 1 and 2):

$$\phi_{ij}(r) = \frac{\phi_{\mathrm{WF}}(r)}{\varepsilon_{ij}} + \phi_{\mathrm{WF}}(r^*) - \frac{\phi_{\mathrm{WF}}(r^*)}{\varepsilon_{ij}} + 2\varepsilon_{ij}(1-\varepsilon_{ij}), \tag{1}$$

$$\phi_{ij}(r) = \begin{cases} -\phi_{\mathrm{WF}}(r^*) - \varepsilon_{ij} & (r < r*) \\ 0 & (r \geq r*) \end{cases}, \tag{2}$$

where $r^* = \sigma \left(\frac{2v + \left[\frac{\sigma}{R_{ij}}\right]^{2\mu}}{1+2v} \right)^{-1/2\mu}$ and $\phi_{\mathrm{WF}}(r)$ is the canonical Wang–Frenkel potential.

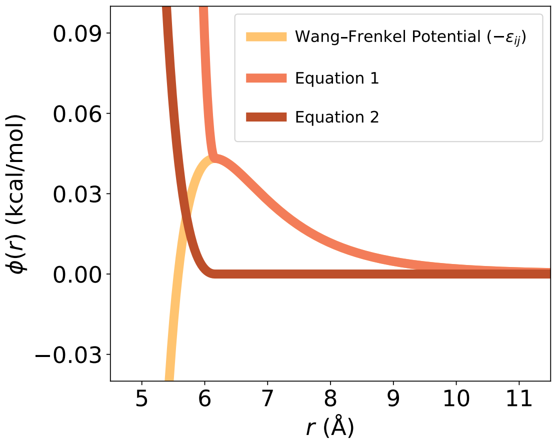

FIG. S1: **New implementations of the Wang–Frenkel potential account for purely repulsive interactions.** Based on the functionals, Equation 2, similar to the WCA potential, preserves the bead size for an amino acid; hence is chosen in the implementation for the Mpipi-T model.

Eq. 1 is similar to the Ashbaugh–Hatch implementation of the Lennard-Jones potential [1], and Eq. 2 is similar to the WCA potential [2]. We choose Eq. 2, since this implementation preserves the molecular diameter (i.e., the bead size).

II. Model Optimization

Optimization of Mpipi-T Models 1, 2, and 3. To develop Mpipi-T, we optimize three distinct models, each employing varying parametrizations of ε in the Wang–Frenkel potential. To this end, the simulated cloud point temperatures (see CLOUD-FIT method in Fig. S9) are trained against experimental data for LCST protein sequences, as reported by Quiroz et al. [3]. These sequences represent a benchmark for modeling LCST phase behavior, providing a robust dataset for parameter optimization.

The cloud point optimization show that all three models achieved RMSD values between 9 K and 13 K (Fig. S2). This level of accuracy is comparable to the

* Corresponding author: jerellejoseph@princeton.edu

performance of the parent Mpipi model, which exhibited an RMSD of 9 K for the critical temperatures extracted from phase diagrams of A1-LCD wild-type and mutant proteins [4, 5]. Model 3 is chosen as the main Mpipi-T model (i.e., for results in the main text) as it has the lowest RMSD value. However, all three models should capture LCST phase behavior reliably. Notably, the largest deviations were observed for ELP sequences with high experimental cloud points. In these cases, the absence of reported error bars or uncertainties in the experimental datasets may contribute to discrepancies, and such variability are worth examining in future model refinements.

III. Model Testing

Testing Mpipi-T Models 1, 2, and 3 show high correlation between simulations with experiments. To further evaluate the accuracy of the Mpipi-T models, single-chain simulations are performed for five ELP sequences using all three optimized models. These simulations are used identify the coil-to-globule transition temperatures (i.e., T_θ) and compare them with the critical temperatures that are estimated experimentally (i.e., T_c) [6]. The sequences of the ELPs tested are as follows:

ELP Variant	Sequence
ELP-1	MSKGPG-$(\text{VGPVG})_{160}$-Y
ELP-2	MSKGPG-$(\text{VPGVGVPGAG})_{40}$-Y
ELP-3	$((\text{VPGVGVPGAG}(\text{VPGVG})_4$-VPGAG$(\text{VPGVG})_3)$-GKG$)_8$-Y
ELP-4	MSKGPG-$(\text{VPGAG})_{80}$-Y
ELP-5	MSKGPG-$(\text{VPGVG})_{40}$-Y

TABLE S1: ELP Sequences Used for Testing

Given that some of these sequences are up to 800 residues long, direct coexistence simulations to compute phase diagrams require extensive simulation times to achieve proper equilibration. Instead, single-chain simulations are first utilized as a proxy for multi-chain simulations.

Across all three models, the simulated coil-to-globule transition temperatures exhibit a high Pearson correlation with the critical temperatures that are estimated experimentally, with correlation coefficients exceeding 0.98 (Fig. S3). Among the three models, Model 3 demonstrates the highest Pearson correlation, highlighting its superior ability in capturing the relationship between sequence and T_θ for ELP sequences.

Computation of phase diagrams for ELP sequences to evaluate the performance of the three Mpipi-T models. Direct coexistence simulations are used to compute phase diagrams of the five ELP sequences. The Pearson correlation coefficients between the critical temperatures predicted by the models and the estimated values from experimental measurements are high: 0.958 for Model 1, 0.986 for Model 2, and 0.932 for Model 3. However, the root-mean-square deviation (RMSD) values reveal greater variability: 16.2 K for Model 1 (Fig. S4a, Fig. S5), 12.4 K for Model 2 (Fig. S4b, Fig. S6), and 18.9 K for Model 3 (Fig. S4c, Fig. S7). It is important to note that we estimate the experimental critical temperature by extrapolating the left arm of the binodal, as this is the available data from experiments [6].

ELP-1, which is more than 800 residues long, is a major contributor to the higher RMSD values. The large size of this sequence makes it challenging to collect reliable statistics, as achieving equilibrium in multi-chain simulations for such a system is expensive computationally. Additionally, the critical temperature of ELP-1, estimated at approximately 295 K from experiments, is relatively low. Simulating such low temperatures requires even longer equilibration times due to slow dynamics, further complicating the reliability of the predictions.

Another factor influencing the final RMSD values is our effort to avoid overfitting. Experimental data can vary between trials; however, we only have access to the average or one of the trials. Furthermore, the experimental data used to test the models are sourced from a study different from the data used for parameter optimization. Variations in experimental methodologies may introduce discrepancies in critical temperature measurements, reflecting their inherent variability.

Despite these challenges, the high Pearson correlation values indicate that the Mpipi-T models capture the overall trends in phase behavior for the ELP sequences effectively. Here, Model 2 performs best, balancing accuracy and reliability, as evidenced by its highest Pearson correlation and lowest RMSD values.

Overall, these results demonstrate that all three Mpipi-T models are useful, but their suitability may depend on the context and the specific system being simulated. For example, Model 3 performs best in capturing LCST behavior across a broad range of disordered sequences and describing the coil-to-globule transition of long ELPs. However, Model 2 shows slightly better performance in reproducing experimental phase diagrams of long ELPs (test here). In summary, we encourage users to test all three models and select the Mpipi-T model that best suits their simulation needs.

Testing model performance under varying experimental conditions. To further evaluate the generalizability of our model, we tested its ability to capture temperature–driven trends for the N-terminal domain

of CPEB4 (448 residues) and several experimentally characterized variants [7]. In these variants, 25% or 50% of histidine residues were substituted with serine, either uniformly across the sequence (H25S and H50S) or within a histidine-rich cluster (H25SHC and H50SHC). Experimental measurements reported cloud point temperatures for each of these sequences at pH 8.

Given the computational cost of multi-chain simulations for such large proteins, we instead used single-chain simulations to estimate the coil-to-globule transition temperatures and compared them to the experimental cloud points. Our model reproduced the correct ordering of cloud points across the five variants, with a Pearson correlation coefficient of 0.83 (Fig. S8). However, the simulated differences in transition temperatures were less pronounced than those observed experimentally.

We attribute this partial discrepancy in part to differences in solution pH: while experiments were conducted at pH 8, our model was developed and parameterized under conditions closer to phosphate-buffered saline (PBS), around pH 7.4. This difference may influence the protonation state and interactions of titratable residues—particularly histidine, which is known to exhibit a chemical shift in this pH range as reported in the experimental study [7]. Incorporating pH sensitivity into future versions of the model may improve its predictive accuracy, especially for systems where titratable residues play a central role in driving phase behavior.

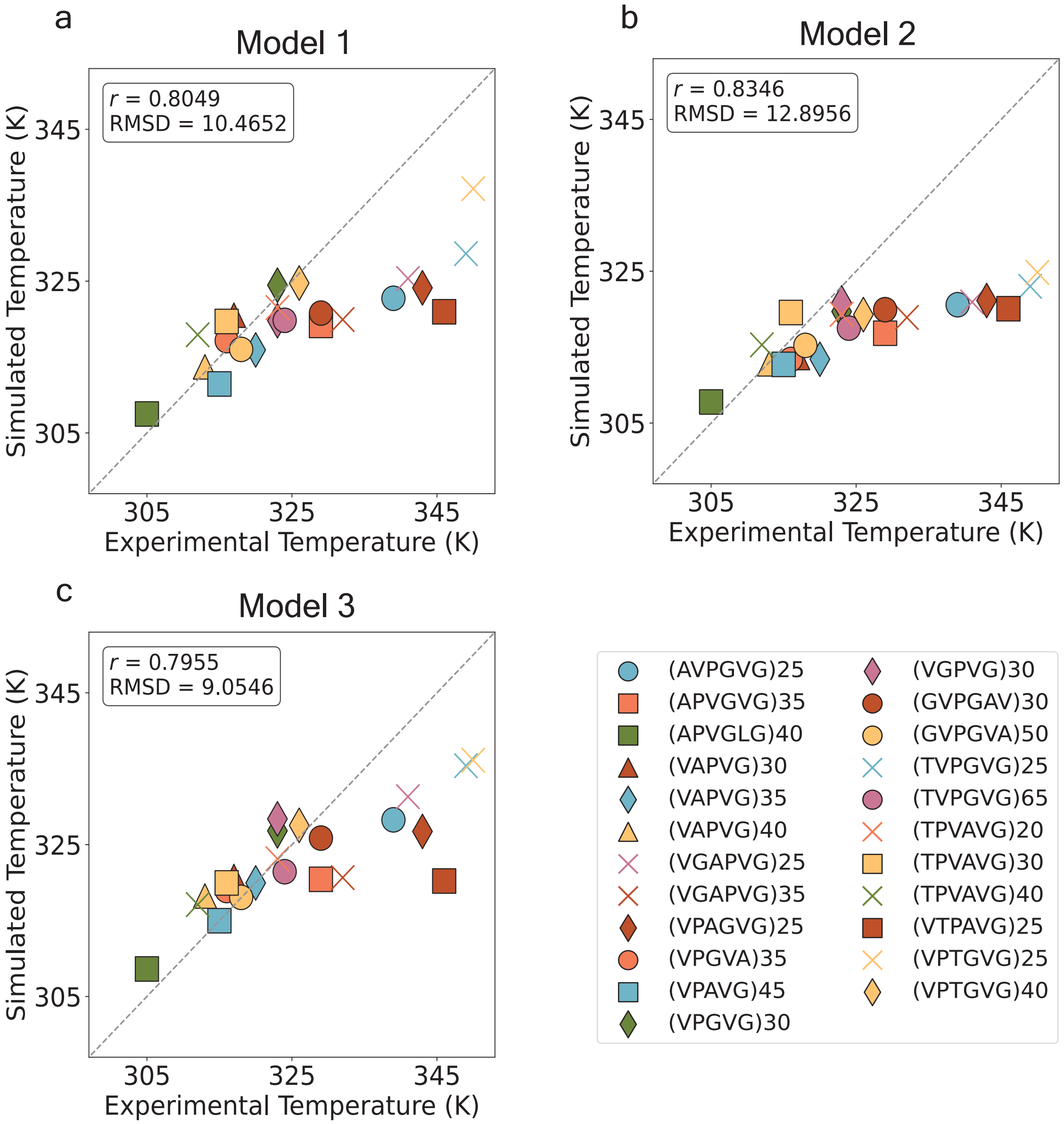

FIG. S2: **Cloud point data from optimization of Mpipi-T for** (a) Model 1, (b) Model 2, and (c) Model 3. The dataset is divided into three blocks, with the mean of the blocks used to compute the data points. The error bars, evaluated as the standard error, are not shown as they are smaller than the size of the data points. The legend, shown in the lower right panel, lists each protein sequence that was simulated. Model 3 is chosen due to its lowest value for RMSD between simulated and experimental cloud point temperatures.

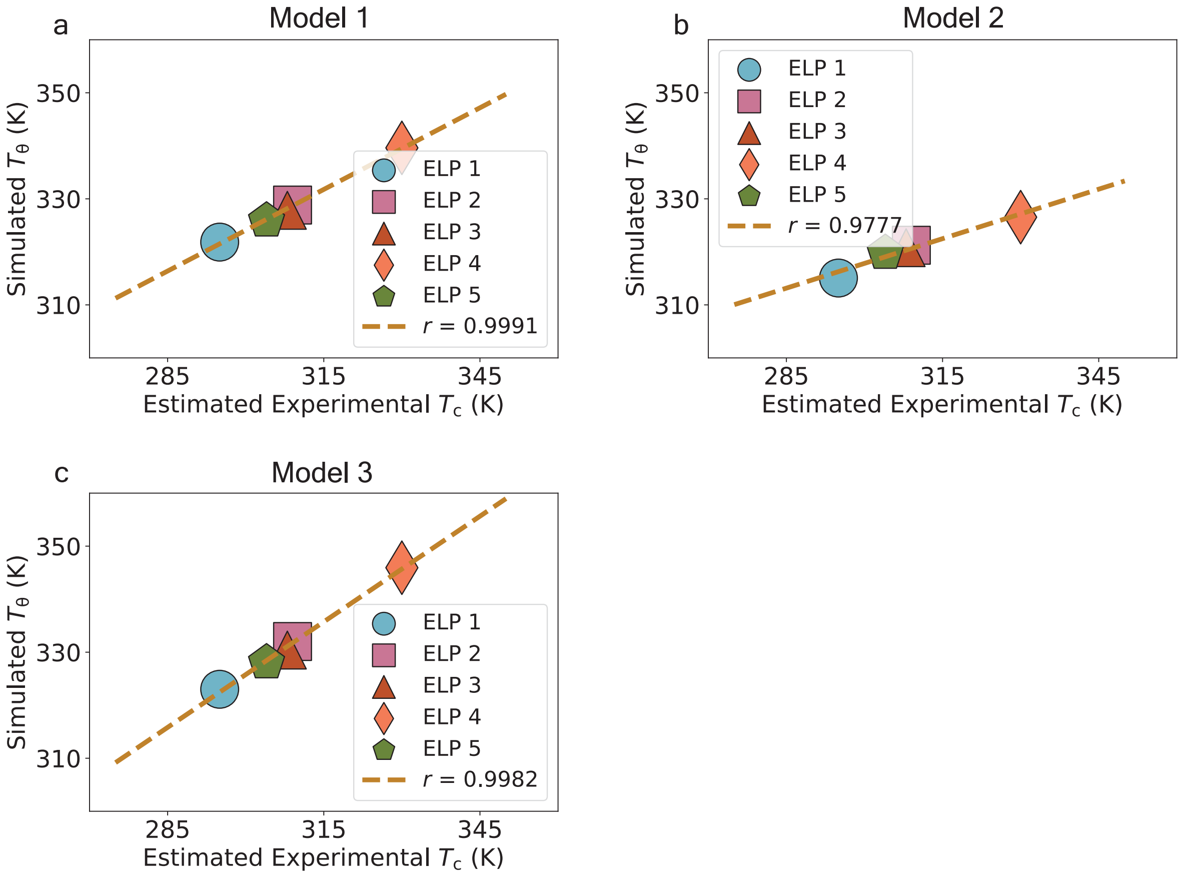

FIG. S3: **Testing of Mpipi-T models by comparing simulated single chain coil-to-globule transition to estimated critical temperature for:** (a) Model 1, (b) Model 2, and (c) Model 3. The dataset is divided into three blocks, with the mean of the blocks computed to determine the data points. The error bars, evaluated using the standard error, are not shown as they are smaller than the size of the data points. The brown line represents the line of best fit. All 3 models perform well, reflecting high Pearson correlation values.

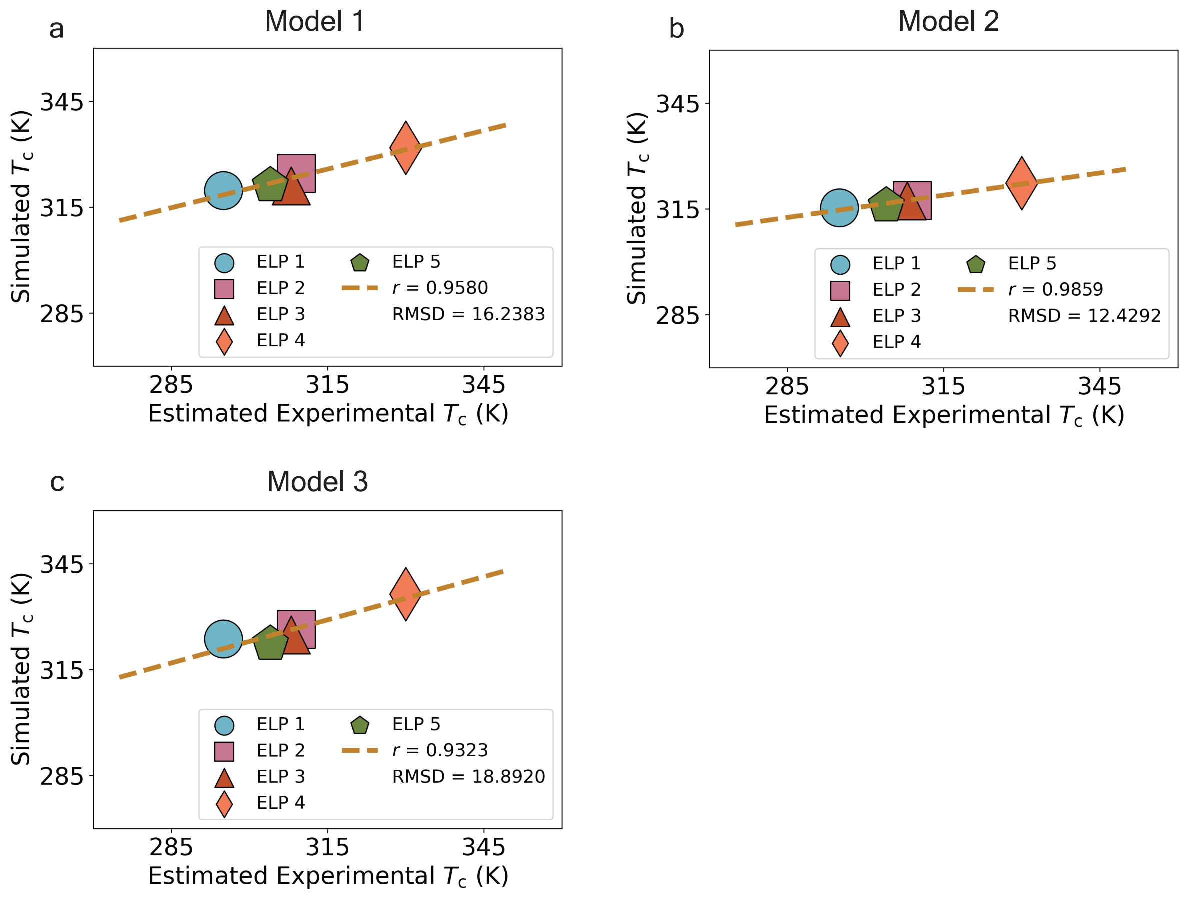

FIG. S4: **Testing of Mpipi-T models by comparing simulated critical temperature to estimated experimental critical temperature for:** (a) Model 1, (b) Model 2, and (c) Model 3. The dataset is divided into three blocks, with the mean of the blocks computed to determine the data points. The error bars, evaluated using the standard error, are not shown as they are smaller than the size of the data points. The brown line represents the line of best fit, and the Pearson correlation and RMSD are shown in the inset. All 3 models perform well, reflecting high Pearson correlation values.

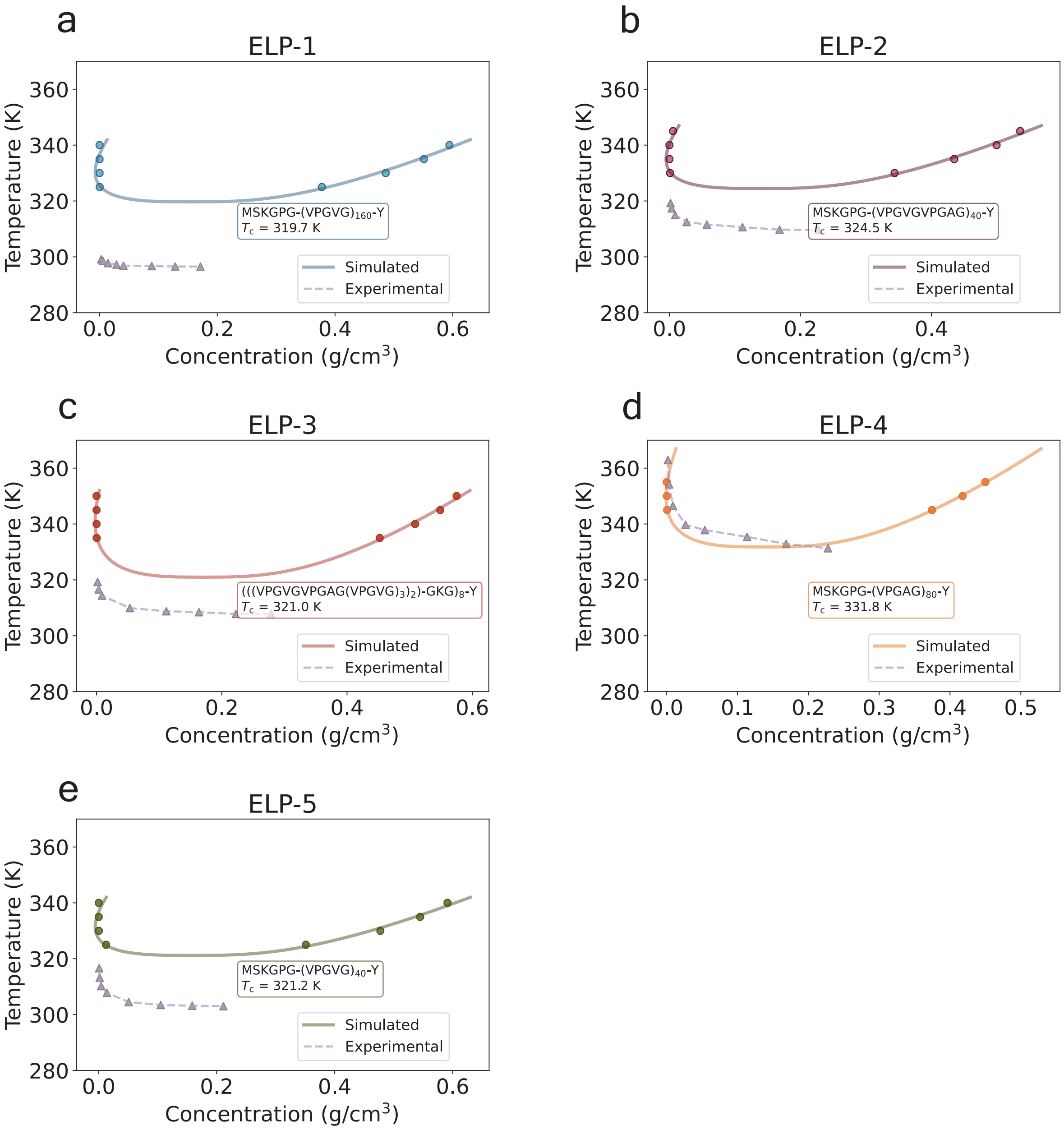

FIG. S5: **Mpipi-T Model 1 phase diagrams computed for:** (a) ELP-1, (b) ELP-2, (c) ELP-3, (d) ELP-4, and (e) ELP-5. The ELP sequence and the critical temperature extracted from simulations (using the law of coexistence densities and law of rectilinear diameters) are shown in the inset. Each trajectory is divided into three blocks, with the mean of the blocks used to determine each data point. The error bars, representing the standard error, are not shown as they are smaller than the size of the data points.

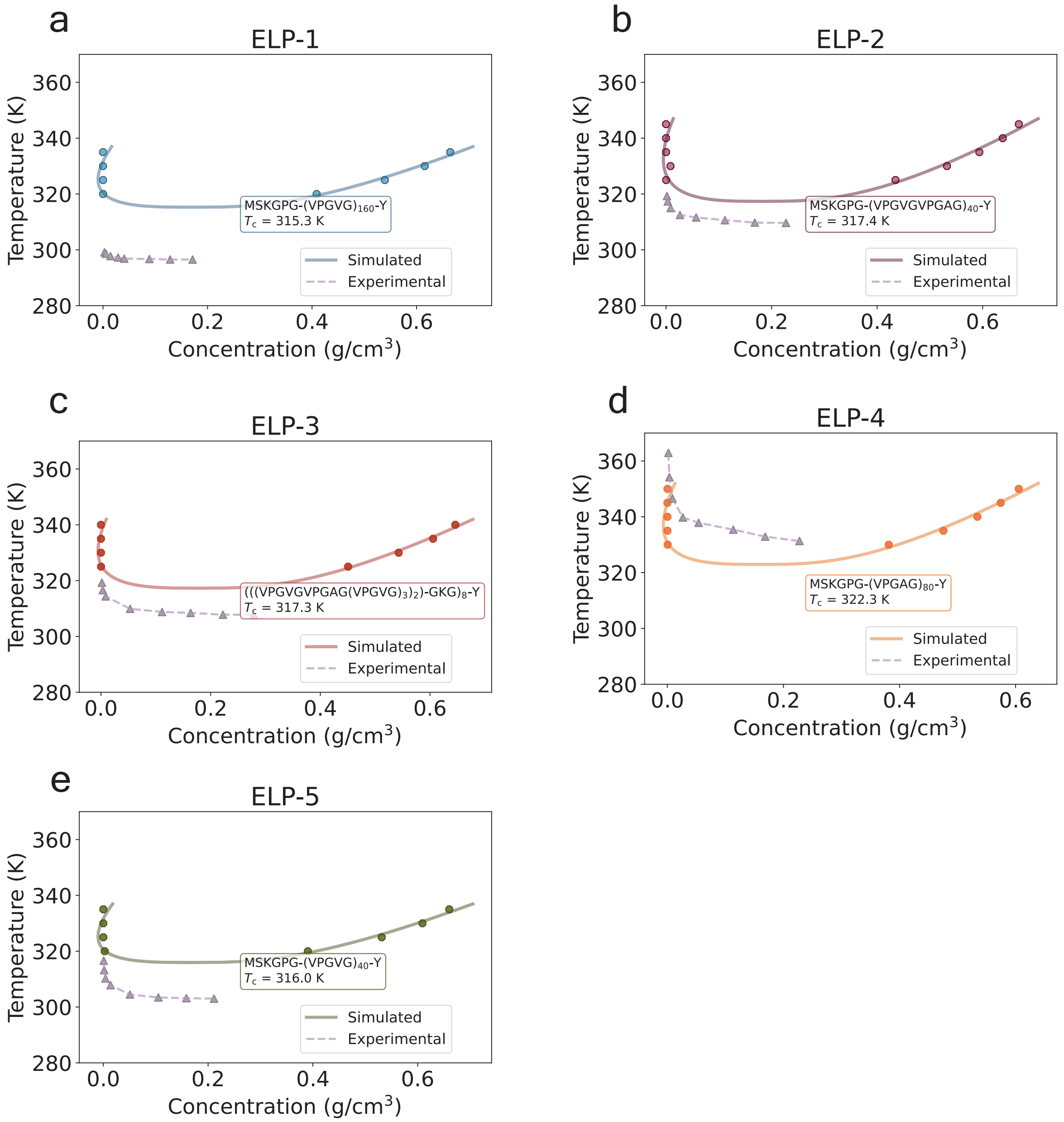

FIG. S6: **Mpipi-T Model 2 phase diagrams computed for:** (a) ELP-1, (b) ELP-2, (c) ELP-3, (d) ELP-4, and (e) ELP-5. The ELP sequence and the critical temperature extracted from simulations (using the law of coexistence densities and law of rectilinear diameters) are shown in the inset. Each trajectory is divided into three blocks, with the mean of the blocks used to determine each data point. The error bars, representing the standard error, are not shown as they are smaller than the size of the data points.

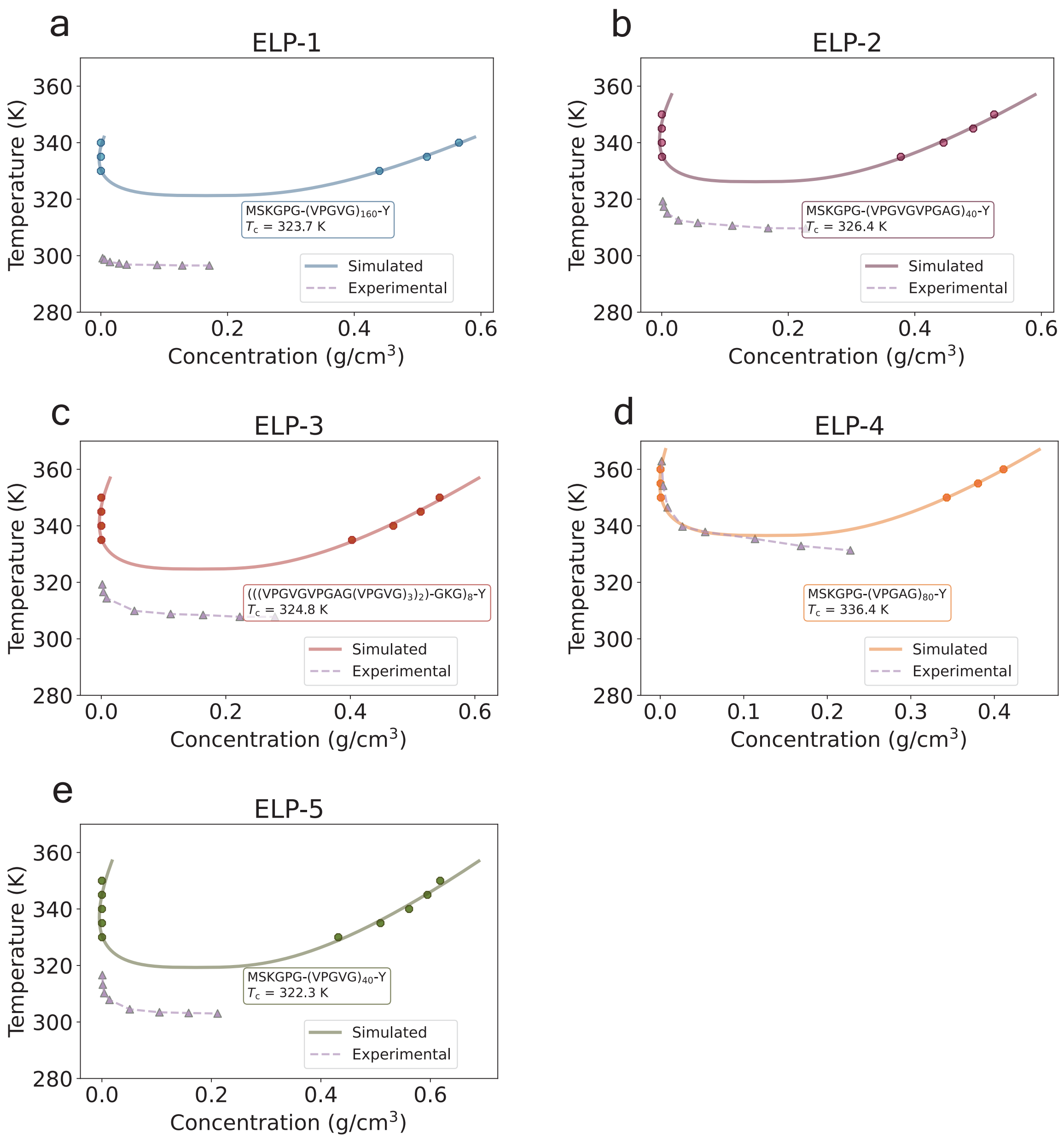

FIG. S7: **Mpipi-T Model 3 phase diagrams computed for:** (a) ELP-1, (b) ELP-2, (c) ELP-3, (d) ELP-4, and (e) ELP-5. The ELP sequence and the critical temperature extracted from simulations (using the law of coexistence densities and law of rectilinear diameters) are shown in the inset. Each trajectory is divided into three blocks, with the mean of the blocks used to determine each data point. The error bars, representing the standard error, are not shown as they are smaller than the size of the data points.

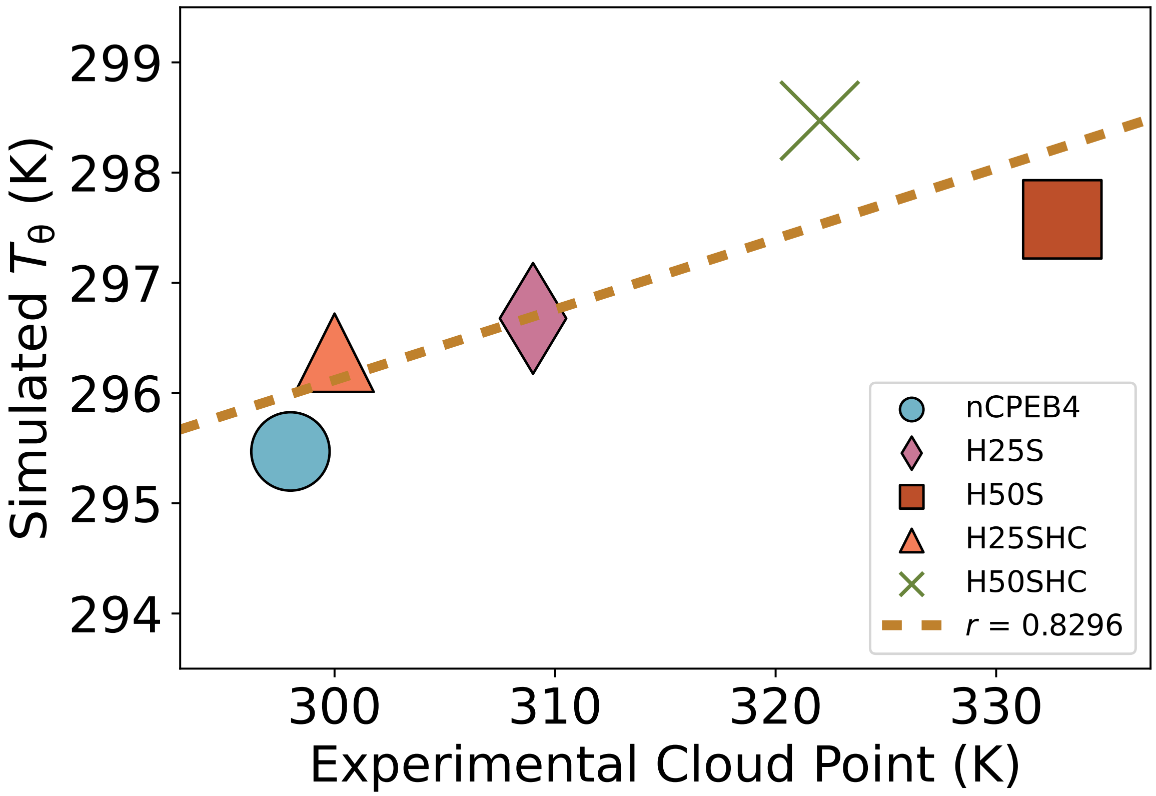

FIG. S8: **Comparison of simulated and experimental temperature dependence across CPEB4 variants.** Simulated coil-to-globule transition temperatures for the N-terminal domain of wild-type CPEB4 (nCPEB4) and four histidine-to-serine variants are compared against experimental cloud point temperatures. The model reproduces the correct ordering of transition temperatures across variants, with a Pearson correlation coefficient of 0.8296, though the magnitude of differences is more subtle in simulations. Discrepancies are likely due in part to pH differences between experimental (pH 8) and simulation (pH 7.4) conditions. The dataset is divided into three blocks, with the mean of the blocks computed to determine the data points. The error bars, evaluated using the standard error, are not shown as they are smaller than the size of the data points. The brown line represents the line of best fit.

IV. CLOUD-FIT: Cloud Point Computations

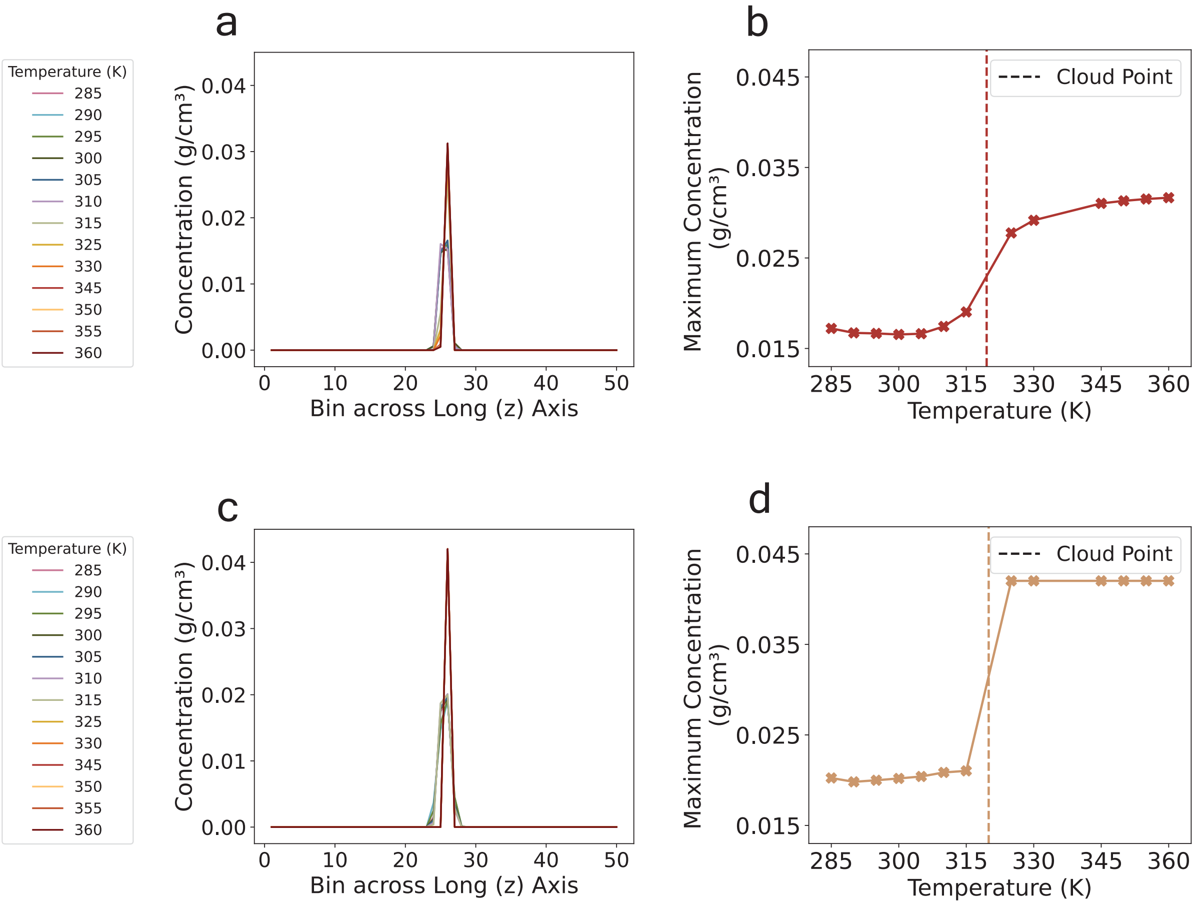

FIG. S9: **CLOUD-FIT: a new method to compute cloud point from simulations in the dilute phase is illustrated using two examples.** At a fixed system concentration, the cloud point is the temperature at which the system becomes turbid due to phase separation. In experiments, this is often measured using a low system concentration. For LCST sequences, the sample is heated gradually from a low system concentration until it reaches the left arm of the binodal. Since nucleation events are rare in dilute solutions, CLOUD-FIT exploits finite-size effects to capture density fluctuations at the target system concentration. A simulation box with proteins is prepared with 64 protein replicates (if protein length is less than 190 residues) or 27 replicates (if protein length is greater than or equal to 190 residues). The box is then compressed using *NPT* simulations to create a slab of high protein density, thereby accelerating the rate at which fluctuations are observed. The z-dimension (or long axis) is extended to achieve the target protein concentration. (a) and (c) show the the density profiles along the z-axis of the slab system after *NVT* simulations are performed for each temperature in small temperature intervals over the desired range. The system is scanned through, and the region of maximum concentration is recorded. The maximum concentration at this region for each of these temperatures in (a) and (c) is extracted and plotted in (b) and (d), respectively. At temperatures below the cloud point, the density is more distributed throughout the box, resulting in a lower maximum concentration. At temperatures above the cloud point, the system condenses, resulting in a higher maximum concentration. The midpoint of the region with the highest slope is determined to be the cloud point.

V. Fitness Function Parameters for Model 3

Tables S2 to S21 are the fitness function parameters for ε_{ij} in Model 3 of Mpipi-T. The parameters for Models 1 and 2 can be found in the Mpipi-T GitHub repository.

Amino Acid i	Amino Acid j	$\varepsilon_{ii,\text{Mpipi}}$	$\varepsilon_{jj,\text{Mpipi}}$	a_i	b_i	c_i	α_i	a_j	b_j	c_j	α_j
A	R	0.049480	0.089916	-6.84807201e-05	5.42590026e-02	-8.46961697e+00	0.70	0	0	0	0
A	H	0.049480	0.027216	-6.84807201e-05	5.42590026e-02	-8.46961697e+00	0.70	0	0	0	0
A	K	0.049480	0.019117	-6.84807201e-05	5.42590026e-02	-8.46961697e+00	0.70	0	0	0	0
A	D	0.049480	0.079096	-6.84807201e-05	5.42590026e-02	-8.46961697e+00	0.70	0	0	0	0
A	E	0.049480	0.085622	-6.84807201e-05	5.42590026e-02	-8.46961697e+00	0.70	0	0	0	0
A	S	0.049480	0.061600	-6.84807201e-05	5.42590026e-02	-8.46961697e+00	0.70	0	0	0	0
A	T	0.049480	0.030758	-6.84807201e-05	5.42590026e-02	-8.46961697e+00	0.70	0	0	0	0
A	N	0.049480	0.193849	-6.84807201e-05	5.42590026e-02	-8.46961697e+00	0.70	0	0	0	0
A	Q	0.049480	0.200448	-6.84807201e-05	5.42590026e-02	-8.46961697e+00	0.70	0	0	0	0
A	C	0.049480	0.069311	-6.84807201e-05	5.42590026e-02	-8.46961697e+00	0.70	0	0	0	0
A	G	0.049480	0.096470	-1.09569152e-04	5.42590026e-02	-8.46961697e+00	0.70	0	0	0	0
A	P	0.049480	0.078677	-6.84807201e-05	5.42590026e-02	-8.46961697e+00	0.70	0	0	0	0
A	A	0.049480	0.049480	-6.84807201e-05	5.42590026e-02	-8.46961697e+00	0.70	-6.84807201e-05	5.42590026e-02	-8.46961697e+00	0.70
A	V	0.049480	0.005578	-6.84807201e-05	5.42590026e-02	-8.46961697e+00	0.70	-1.49763237e-04	1.15406051e-01	-1.92322375e+01	0.70
A	I	0.049480	0.000395	-6.84807201e-05	5.42590026e-02	-8.46961697e+00	0.70	-9.88175293e-05	1.10797972e-01	-1.85218569e+01	0.70
A	L	0.049480	0.010998	-6.84807201e-05	5.42590026e-02	-8.46961697e+00	0.70	-1.06970639e-04	1.17281979e-01	-1.91871215e+01	0.70
A	M	0.049480	0.039564	-6.84807201e-05	5.42590026e-02	-8.46961697e+00	0.70	-9.14334357e-05	8.01341277e-02	-1.76908670e+01	0.70
A	F	0.049480	0.391642	-6.84807201e-05	5.42590026e-02	-8.46961697e+00	0.70	0	0	0	0
A	Y	0.049480	0.419186	-6.84807201e-05	5.42590026e-02	-8.46961697e+00	0.70	0	0	0	0
A	W	0.049480	0.550297	-6.84807201e-05	5.42590026e-02	-8.46961697e+00	0.70	0	0	0	0

TABLE S2: Fitness function parameters for ε_{ij} where i = A in Mpipi-T Model 3

Amino Acid i	Amino Acid j	$\varepsilon_{ii,\text{Mpipi}}$	$\varepsilon_{jj,\text{Mpipi}}$	a_i	b_i	c_i	α_i	a_j	b_j	c_j	α_j
C	R	0.069311	0.089916	0	0	0	0	0	0	0	0
C	H	0.069311	0.027216	0	0	0	0	0	0	0	0
C	K	0.069311	0.019117	0	0	0	0	0	0	0	0
C	D	0.069311	0.079096	0	0	0	0	0	0	0	0
C	E	0.069311	0.085622	0	0	0	0	0	0	0	0
C	S	0.069311	0.061600	0	0	0	0	0	0	0	0
C	T	0.069311	0.030758	0	0	0	0	0	0	0	0
C	N	0.069311	0.193849	0	0	0	0	0	0	0	0
C	Q	0.069311	0.200448	0	0	0	0	0	0	0	0
C	C	0.069311	0.069311	0	0	0	0	0	0	0	0
C	G	0.069311	0.096470	0	0	0	0	0	0	0	0
C	P	0.069311	0.078677	0	0	0	0	0	0	0	0
C	A	0.069311	0.049480	0	0	0	0	-6.84807201e-05	5.42590026e-02	-8.46961697e+00	0.70
C	V	0.069311	0.005578	0	0	0	0	-1.49763237e-04	1.15406051e-01	-1.92322375e+01	0.70
C	I	0.069311	0.000395	0	0	0	0	-9.88175293e-05	1.10797972e-01	-1.85218569e+01	0.70
C	L	0.069311	0.010998	0	0	0	0	-1.06970639e-04	1.17281979e-01	-1.91871215e+01	0.70
C	M	0.069311	0.039564	0	0	0	0	-9.14334357e-05	8.01341277e-02	-1.76908670e+01	0.70
C	F	0.069311	0.391642	0	0	0	0	0	0	0	0
C	Y	0.069311	0.419186	0	0	0	0	0	0	0	0
C	W	0.069311	0.550297	0	0	0	0	0	0	0	0

TABLE S3: Fitness function parameters for ε_{ij} where i = C in Mpipi-T Model 3

Amino Acid i	Amino Acid j	$\varepsilon_{ii,\text{Mpipi}}$	$\varepsilon_{jj,\text{Mpipi}}$	a_i	b_i	c_i	α_i	a_j	b_j	c_j	α_j
D	R	0.079096	0.089916	0	0	0	0	0	0	0	0
D	H	0.079096	0.027216	0	0	0	0	0	0	0	0
D	K	0.079096	0.019117	0	0	0	0	0	0	0	0
D	D	0.079096	0.079096	0	0	0	0	0	0	0	0
D	E	0.079096	0.085622	0	0	0	0	0	0	0	0
D	S	0.079096	0.061600	0	0	0	0	0	0	0	0
D	T	0.079096	0.030758	0	0	0	0	0	0	0	0
D	N	0.079096	0.193849	0	0	0	0	0	0	0	0
D	Q	0.079096	0.200448	0	0	0	0	0	0	0	0
D	C	0.079096	0.069311	0	0	0	0	0	0	0	0
D	G	0.079096	0.096470	0	0	0	0	0	0	0	0
D	P	0.079096	0.078677	0	0	0	0	0	0	0	0
D	A	0.079096	0.049480	0	0	0	0	-6.84807201e-05	5.42590026e-02	-8.46961697e+00	0.70
D	V	0.079096	0.005578	0	0	0	0	-1.49763237e-04	1.15406051e-01	-1.92322375e+01	0.70
D	I	0.079096	0.000395	0	0	0	0	-9.88175293e-05	1.10797972e-01	-1.85218569e+01	0.70
D	L	0.079096	0.010998	0	0	0	0	-1.06970639e-04	1.17281979e-01	-1.91871215e+01	0.70
D	M	0.079096	0.039564	0	0	0	0	-9.14334357e-05	8.01341277e-02	-1.76908670e+01	0.70
D	F	0.079096	0.391642	0	0	0	0	0	0	0	0
D	Y	0.079096	0.419186	0	0	0	0	0	0	0	0
D	W	0.079096	0.550297	0	0	0	0	0	0	0	0

TABLE S4: Fitness function parameters for ε_{ij} where i = D in Mpipi-T Model 3

Amino Acid i	Amino Acid j	$\varepsilon_{ii,\text{Mpipi}}$	$\varepsilon_{jj,\text{Mpipi}}$	a_i	b_i	c_i	α_i	a_j	b_j	c_j	α_j
E	R	0.085622	0.089916	0	0	0	0	0	0	0	0
E	H	0.085622	0.027216	0	0	0	0	0	0	0	0
E	K	0.085622	0.019117	0	0	0	0	0	0	0	0
E	D	0.085622	0.079096	0	0	0	0	0	0	0	0
E	E	0.085622	0.085622	0	0	0	0	0	0	0	0
E	S	0.085622	0.061600	0	0	0	0	0	0	0	0
E	T	0.085622	0.030758	0	0	0	0	0	0	0	0
E	N	0.085622	0.193849	0	0	0	0	0	0	0	0
E	Q	0.085622	0.200448	0	0	0	0	0	0	0	0
E	C	0.085622	0.069311	0	0	0	0	0	0	0	0
E	G	0.085622	0.096470	0	0	0	0	0	0	0	0
E	P	0.085622	0.078677	0	0	0	0	0	0	0	0
E	A	0.085622	0.049480	0	0	0	0	-6.84807201e-05	5.42590026e-02	-8.46961697e+00	0.70
E	V	0.085622	0.005578	0	0	0	0	-1.49763237e-04	1.15406051e-01	-1.92322375e+01	0.70
E	I	0.085622	0.000395	0	0	0	0	-9.88175293e-05	1.10797972e-01	-1.85218569e+01	0.70
E	L	0.085622	0.010998	0	0	0	0	-1.06970639e-04	1.17281979e-01	-1.91871215e+01	0.70
E	M	0.085622	0.039564	0	0	0	0	-9.14334357e-05	8.01341277e-02	-1.76908670e+01	0.70
E	F	0.085622	0.391642	0	0	0	0	0	0	0	0
E	Y	0.085622	0.419186	0	0	0	0	0	0	0	0
E	W	0.085622	0.550297	0	0	0	0	0	0	0	0

TABLE S5: Fitness function parameters for ε_{ij} where i = E in Mpipi-T Model 3

Amino Acid i	Amino Acid j	$\varepsilon_{ii,\mathrm{Mpipi}}$	$\varepsilon_{jj,\mathrm{Mpipi}}$	a_i	b_i	c_i	α_i	a_j	b_j	c_j	α_j
F	R	0.391642	0.089916	0	0	0	0	0	0	0	0
F	H	0.391642	0.027216	0	0	0	0	0	0	0	0
F	K	0.391642	0.019117	0	0	0	0	0	0	0	0
F	D	0.391642	0.079096	0	0	0	0	0	0	0	0
F	E	0.391642	0.085622	0	0	0	0	0	0	0	0
F	S	0.391642	0.061600	0	0	0	0	0	0	0	0
F	T	0.391642	0.030758	0	0	0	0	0	0	0	0
F	N	0.391642	0.193849	0	0	0	0	0	0	0	0
F	Q	0.391642	0.200448	0	0	0	0	0	0	0	0
F	C	0.391642	0.069311	0	0	0	0	0	0	0	0
F	G	0.391642	0.096470	0	0	0	0	0	0	0	0
F	P	0.391642	0.078677	0	0	0	0	0	0	0	0
F	A	0.391642	0.049480	0	0	0	0	-6.84807201e-05	5.42590026e-02	-8.46961697e+00	0.70
F	V	0.391642	0.005578	0	0	0	0	-1.49763237e-04	1.15406051e-01	-1.92322375e+01	0.70
F	I	0.391642	0.000395	0	0	0	0	-9.88175293e-05	1.10797972e-01	-1.85218569e+01	0.70
F	L	0.391642	0.010998	0	0	0	0	-1.06970639e-04	1.17281979e-01	-1.91871215e+01	0.70
F	M	0.391642	0.039564	0	0	0	0	-9.14334357e-05	8.01341277e-02	-1.76908670e+01	0.70
F	F	0.391642	0.391642	0	0	0	0	0	0	0	0
F	Y	0.391642	0.419186	0	0	0	0	0	0	0	0
F	W	0.391642	0.550297	0	0	0	0	0	0	0	0

TABLE S6: Fitness function parameters for ε_{ij} where i = F in Mpipi-T Model 3

Amino Acid i	Amino Acid j	$\varepsilon_{ii,\mathrm{Mpipi}}$	$\varepsilon_{jj,\mathrm{Mpipi}}$	a_i	b_i	c_i	α_i	a_j	b_j	c_j	α_j
G	R	0.096470	0.089916	0	0	0	0	0	0	0	0
G	H	0.096470	0.027216	0	0	0	0	0	0	0	0
G	K	0.096470	0.019117	0	0	0	0	0	0	0	0
G	D	0.096470	0.079096	0	0	0	0	0	0	0	0
G	E	0.096470	0.085622	0	0	0	0	0	0	0	0
G	S	0.096470	0.061600	0	0	0	0	0	0	0	0
G	T	0.096470	0.030758	0	0	0	0	0	0	0	0
G	N	0.096470	0.193849	0	0	0	0	0	0	0	0
G	Q	0.096470	0.200448	0	0	0	0	0	0	0	0
G	C	0.096470	0.069311	0	0	0	0	0	0	0	0
G	G	0.096470	0.096470	0	0	0	0	0	0	0	0
G	P	0.096470	0.078677	0	0	0	0	0	0	0	0
G	A	0.096470	0.049480	0	0	0	0	-1.09569152e-04	5.42590026e-02	-8.46961697e+00	0.70
G	V	0.096470	0.005578	0	0	0	0	-2.69573827e-04	1.15406051e-01	-1.92322375e+01	0.70
G	I	0.096470	0.000395	0	0	0	0	-1.77871553e-04	1.10797972e-01	-1.85218569e+01	0.70
G	L	0.096470	0.010998	0	0	0	0	-1.92547149e-04	1.17281979e-01	-1.91871215e+01	0.70
G	M	0.096470	0.039564	0	0	0	0	-1.64580184e-04	8.01341277e-02	-1.76908670e+01	0.70
G	F	0.096470	0.391642	0	0	0	0	0	0	0	0
G	Y	0.096470	0.419186	0	0	0	0	0	0	0	0
G	W	0.096470	0.550297	0	0	0	0	0	0	0	0

TABLE S7: Fitness function parameters for ε_{ij} where i = G in Mpipi-T Model 3

Amino Acid i	Amino Acid j	$\varepsilon_{ii,\mathrm{Mpipi}}$	$\varepsilon_{jj,\mathrm{Mpipi}}$	a_i	b_i	c_i	α_i	a_j	b_j	c_j	α_j
H	R	0.027216	0.089916	0	0	0	0	0	0	0	0
H	H	0.027216	0.027216	0	0	0	0	0	0	0	0
H	K	0.027216	0.019117	0	0	0	0	0	0	0	0
H	D	0.027216	0.079096	0	0	0	0	0	0	0	0
H	E	0.027216	0.085622	0	0	0	0	0	0	0	0
H	S	0.027216	0.061600	0	0	0	0	0	0	0	0
H	T	0.027216	0.030758	0	0	0	0	0	0	0	0
H	N	0.027216	0.193849	0	0	0	0	0	0	0	0
H	Q	0.027216	0.200448	0	0	0	0	0	0	0	0
H	C	0.027216	0.069311	0	0	0	0	0	0	0	0
H	G	0.027216	0.096470	0	0	0	0	0	0	0	0
H	P	0.027216	0.078677	0	0	0	0	0	0	0	0
H	A	0.027216	0.049480	0	0	0	0	-6.84807201e-05	5.42590026e-02	-8.46961697e+00	0.70
H	V	0.027216	0.005578	0	0	0	0	-1.49763237e-04	1.15406051e-01	-1.92322375e+01	0.70
H	I	0.027216	0.000395	0	0	0	0	-9.88175293e-05	1.10797972e-01	-1.85218569e+01	0.70
H	L	0.027216	0.010998	0	0	0	0	-1.06970639e-04	1.17281979e-01	-1.91871215e+01	0.70
H	M	0.027216	0.039564	0	0	0	0	-9.14334357e-05	8.01341277e-02	-1.76908670e+01	0.70
H	F	0.027216	0.391642	0	0	0	0	0	0	0	0
H	Y	0.027216	0.419186	0	0	0	0	0	0	0	0
H	W	0.027216	0.550297	0	0	0	0	0	0	0	0

TABLE S8: Fitness function parameters for ε_{ij} where i = H in Mpipi-T Model 3

Amino Acid i	Amino Acid j	$\varepsilon_{ii,\mathrm{Mpipi}}$	$\varepsilon_{jj,\mathrm{Mpipi}}$	a_i	b_i	c_i	α_i	a_j	b_j	c_j	α_j
I	R	0.000395	0.089916	-9.88175293e-05	1.10797972e-01	-1.85218569e+01	0.70	0	0	0	0
I	H	0.000395	0.027216	-9.88175293e-05	1.10797972e-01	-1.85218569e+01	0.70	0	0	0	0
I	K	0.000395	0.019117	-9.88175293e-05	1.10797972e-01	-1.85218569e+01	0.70	0	0	0	0
I	D	0.000395	0.079096	-9.88175293e-05	1.10797972e-01	-1.85218569e+01	0.70	0	0	0	0
I	E	0.000395	0.085622	-9.88175293e-05	1.10797972e-01	-1.85218569e+01	0.70	0	0	0	0
I	S	0.000395	0.061600	-9.88175293e-05	1.10797972e-01	-1.85218569e+01	0.70	0	0	0	0
I	T	0.000395	0.030758	-9.88175293e-05	1.10797972e-01	-1.85218569e+01	0.70	0	0	0	0
I	N	0.000395	0.193849	-9.88175293e-05	1.10797972e-01	-1.85218569e+01	0.70	0	0	0	0
I	Q	0.000395	0.200448	-9.88175293e-05	1.10797972e-01	-1.85218569e+01	0.70	0	0	0	0
I	C	0.000395	0.069311	-9.88175293e-05	1.10797972e-01	-1.85218569e+01	0.70	0	0	0	0
I	G	0.000395	0.096470	-1.77871553e-04	1.10797972e-01	-1.85218569e+01	0.70	0	0	0	0
I	P	0.000395	0.078677	-9.88175293e-05	1.10797972e-01	-1.85218569e+01	0.70	0	0	0	0
I	A	0.000395	0.049480	-9.88175293e-05	1.10797972e-01	-1.85218569e+01	0.70	-6.84807201e-05	5.42590026e-02	-8.46961697e+00	0.70
I	V	0.000395	0.005578	-9.88175293e-05	1.10797972e-01	-1.85218569e+01	0.70	-1.49763237e-04	1.15406051e-01	-1.92322375e+01	0.70
I	I	0.000395	0.000395	-9.88175293e-05	1.10797972e-01	-1.85218569e+01	0.70	-9.88175293e-05	1.10797972e-01	-1.85218569e+01	0.70
I	L	0.000395	0.010998	-9.88175293e-05	1.10797972e-01	-1.85218569e+01	0.70	-1.06970639e-04	1.17281979e-01	-1.91871215e+01	0.70
I	M	0.000395	0.039564	-9.88175293e-05	1.10797972e-01	-1.85218569e+01	0.70	-9.14334357e-05	8.01341277e-02	-1.76908670e+01	0.70
I	F	0.000395	0.391642	-9.88175293e-05	1.10797972e-01	-1.85218569e+01	0.70	0	0	0	0
I	Y	0.000395	0.419186	-9.88175293e-05	1.10797972e-01	-1.85218569e+01	0.70	0	0	0	0
I	W	0.000395	0.550297	-9.88175293e-05	1.10797972e-01	-1.85218569e+01	0.70	0	0	0	0

TABLE S9: Fitness function parameters for ε_{ij} where i = I in Mpipi-T Model 3

Amino Acid i	Amino Acid j	$\varepsilon_{ii,\mathrm{Mpipi}}$	$\varepsilon_{jj,\mathrm{Mpipi}}$	a_i	b_i	c_i	α_i	a_j	b_j	c_j	α_j
K	R	0.019117	0.089916	0	0	0	0	0	0	0	0
K	H	0.019117	0.027216	0	0	0	0	0	0	0	0
K	K	0.019117	0.019117	0	0	0	0	0	0	0	0
K	D	0.019117	0.079096	0	0	0	0	0	0	0	0
K	E	0.019117	0.085622	0	0	0	0	0	0	0	0
K	S	0.019117	0.061600	0	0	0	0	0	0	0	0
K	T	0.019117	0.030758	0	0	0	0	0	0	0	0
K	N	0.019117	0.193849	0	0	0	0	0	0	0	0
K	Q	0.019117	0.200448	0	0	0	0	0	0	0	0
K	C	0.019117	0.069311	0	0	0	0	0	0	0	0
K	G	0.019117	0.096470	0	0	0	0	0	0	0	0
K	P	0.019117	0.078677	0	0	0	0	0	0	0	0
K	A	0.019117	0.049480	0	0	0	0	-6.84807201e-05	5.42590026e-02	-8.46961697e+00	0.70
K	V	0.019117	0.005578	0	0	0	0	-1.49763237e-04	1.15406051e-01	-1.92322375e+01	0.70
K	I	0.019117	0.000395	0	0	0	0	-9.88175293e-05	1.10797972e-01	-1.85218569e+01	0.70
K	L	0.019117	0.010998	0	0	0	0	-1.06970639e-04	1.17281979e-01	-1.91871215e+01	0.70
K	M	0.019117	0.039564	0	0	0	0	-9.14334357e-05	8.01341277e-02	-1.76908670e+01	0.70
K	F	0.019117	0.391642	0	0	0	0	0	0	0	0
K	Y	0.019117	0.419186	0	0	0	0	0	0	0	0
K	W	0.019117	0.550297	0	0	0	0	0	0	0	0

TABLE S10: Fitness function parameters for ε_{ij} where i = K in Mpipi-T Model 3

Amino Acid i	Amino Acid j	$\varepsilon_{ii,\mathrm{Mpipi}}$	$\varepsilon_{jj,\mathrm{Mpipi}}$	a_i	b_i	c_i	α_i	a_j	b_j	c_j	α_j
L	R	0.010998	0.089916	-1.06970639e-04	1.17281979e-01	-1.91871215e+01	0.70	0	0	0	0
L	H	0.010998	0.027216	-1.06970639e-04	1.17281979e-01	-1.91871215e+01	0.70	0	0	0	0
L	K	0.010998	0.019117	-1.06970639e-04	1.17281979e-01	-1.91871215e+01	0.70	0	0	0	0
L	D	0.010998	0.079096	-1.06970639e-04	1.17281979e-01	-1.91871215e+01	0.70	0	0	0	0
L	E	0.010998	0.085622	-1.06970639e-04	1.17281979e-01	-1.91871215e+01	0.70	0	0	0	0
L	S	0.010998	0.061600	-1.06970639e-04	1.17281979e-01	-1.91871215e+01	0.70	0	0	0	0
L	T	0.010998	0.030758	-1.06970639e-04	1.17281979e-01	-1.91871215e+01	0.70	0	0	0	0
L	N	0.010998	0.193849	-1.06970639e-04	1.17281979e-01	-1.91871215e+01	0.70	0	0	0	0
L	Q	0.010998	0.200448	-1.06970639e-04	1.17281979e-01	-1.91871215e+01	0.70	0	0	0	0
L	C	0.010998	0.069311	-1.06970639e-04	1.17281979e-01	-1.91871215e+01	0.70	0	0	0	0
L	G	0.010998	0.096470	-1.92547149e-04	1.17281979e-01	-1.91871215e+01	0.70	0	0	0	0
L	P	0.010998	0.078677	-1.06970639e-04	1.17281979e-01	-1.91871215e+01	0.70	0	0	0	0
L	A	0.010998	0.049480	-1.06970639e-04	1.17281979e-01	-1.91871215e+01	0.70	-6.84807201e-05	5.42590026e-02	-8.46961697e+00	0.70
L	V	0.010998	0.005578	-1.06970639e-04	1.17281979e-01	-1.91871215e+01	0.70	-1.49763237e-04	1.15406051e-01	-1.92322375e+01	0.70
L	I	0.010998	0.000395	-1.06970639e-04	1.17281979e-01	-1.91871215e+01	0.70	-9.88175293e-05	1.10797972e-01	-1.85218569e+01	0.70
L	L	0.010998	0.010998	-1.06970639e-04	1.17281979e-01	-1.91871215e+01	0.70	-1.06970639e-04	1.17281979e-01	-1.91871215e+01	0.70
L	M	0.010998	0.039564	-1.06970639e-04	1.17281979e-01	-1.91871215e+01	0.70	-9.14334357e-05	8.01341277e-02	-1.76908670e+01	0.70
L	F	0.010998	0.391642	-1.06970639e-04	1.17281979e-01	-1.91871215e+01	0.70	0	0	0	0
L	Y	0.010998	0.419186	-1.06970639e-04	1.17281979e-01	-1.91871215e+01	0.70	0	0	0	0
L	W	0.010998	0.550297	-1.06970639e-04	1.17281979e-01	-1.91871215e+01	0.70	0	0	0	0

TABLE S11: Fitness function parameters for ε_{ij} where i = L in Mpipi-T Model 3

Amino Acid i	Amino Acid j	$\varepsilon_{ii,\mathrm{Mpipi}}$	$\varepsilon_{jj,\mathrm{Mpipi}}$	a_i	b_i	c_i	α_i	a_j	b_j	c_j	α_j
M	R	0.039564	0.089916	-9.14334357e-05	8.01341277e-02	-1.76908670e+01	0.70	0	0	0	0
M	H	0.039564	0.027216	-9.14334357e-05	8.01341277e-02	-1.76908670e+01	0.70	0	0	0	0
M	K	0.039564	0.019117	-9.14334357e-05	8.01341277e-02	-1.76908670e+01	0.70	0	0	0	0
M	D	0.039564	0.079096	-9.14334357e-05	8.01341277e-02	-1.76908670e+01	0.70	0	0	0	0
M	E	0.039564	0.085622	-9.14334357e-05	8.01341277e-02	-1.76908670e+01	0.70	0	0	0	0
M	S	0.039564	0.061600	-9.14334357e-05	8.01341277e-02	-1.76908670e+01	0.70	0	0	0	0
M	T	0.039564	0.030758	-9.14334357e-05	8.01341277e-02	-1.76908670e+01	0.70	0	0	0	0
M	N	0.039564	0.193849	-9.14334357e-05	8.01341277e-02	-1.76908670e+01	0.70	0	0	0	0
M	Q	0.039564	0.200448	-9.14334357e-05	8.01341277e-02	-1.76908670e+01	0.70	0	0	0	0
M	C	0.039564	0.069311	-9.14334357e-05	8.01341277e-02	-1.76908670e+01	0.70	0	0	0	0
M	G	0.039564	0.096470	-1.64580184e-04	8.01341277e-02	-1.76908670e+01	0.70	0	0	0	0
M	P	0.039564	0.078677	-9.14334357e-05	8.01341277e-02	-1.76908670e+01	0.70	0	0	0	0
M	A	0.039564	0.049480	-9.14334357e-05	8.01341277e-02	-1.76908670e+01	0.70	-6.84807201e-05	5.42590026e-02	-8.46961697e+00	0.70
M	V	0.039564	0.005578	-9.14334357e-05	8.01341277e-02	-1.76908670e+01	0.70	-1.49763237e-04	1.15406051e-01	-1.92322375e+01	0.70
M	I	0.039564	0.000395	-9.14334357e-05	8.01341277e-02	-1.76908670e+01	0.70	-9.88175293e-05	1.10797972e-01	-1.85218569e+01	0.70
M	L	0.039564	0.010998	-9.14334357e-05	8.01341277e-02	-1.76908670e+01	0.70	-1.06970639e-04	1.17281979e-01	-1.91871215e+01	0.70
M	M	0.039564	0.039564	-9.14334357e-05	8.01341277e-02	-1.76908670e+01	0.70	-9.14334357e-05	8.01341277e-02	-1.76908670e+01	0.70
M	F	0.039564	0.391642	-9.14334357e-05	8.01341277e-02	-1.76908670e+01	0.70	0	0	0	0
M	Y	0.039564	0.419186	-9.14334357e-05	8.01341277e-02	-1.76908670e+01	0.70	0	0	0	0
M	W	0.039564	0.550297	-9.14334357e-05	8.01341277e-02	-1.76908670e+01	0.70	0	0	0	0

TABLE S12: Fitness function parameters for ε_{ij} where i = M in Mpipi-T Model 3

Amino Acid i	Amino Acid j	$\varepsilon_{ii,\mathrm{Mpipi}}$	$\varepsilon_{jj,\mathrm{Mpipi}}$	a_i	b_i	c_i	α_i	a_j	b_j	c_j	α_j
N	R	0.193849	0.089916	0	0	0	0	0	0	0	0
N	H	0.193849	0.027216	0	0	0	0	0	0	0	0
N	K	0.193849	0.019117	0	0	0	0	0	0	0	0
N	D	0.193849	0.079096	0	0	0	0	0	0	0	0
N	E	0.193849	0.085622	0	0	0	0	0	0	0	0
N	S	0.193849	0.061600	0	0	0	0	0	0	0	0
N	T	0.193849	0.030758	0	0	0	0	0	0	0	0
N	N	0.193849	0.193849	0	0	0	0	0	0	0	0
N	Q	0.193849	0.200448	0	0	0	0	0	0	0	0
N	C	0.193849	0.069311	0	0	0	0	0	0	0	0
N	G	0.193849	0.096470	0	0	0	0	0	0	0	0
N	P	0.193849	0.078677	0	0	0	0	0	0	0	0
N	A	0.193849	0.049480	0	0	0	0	-6.84807201e-05	5.42590026e-02	-8.46961697e+00	0.70
N	V	0.193849	0.005578	0	0	0	0	-1.49763237e-04	1.15406051e-01	-1.92322375e+01	0.70
N	I	0.193849	0.000395	0	0	0	0	-9.88175293e-05	1.10797972e-01	-1.85218569e+01	0.70
N	L	0.193849	0.010998	0	0	0	0	-1.06970639e-04	1.17281979e-01	-1.91871215e+01	0.70
N	M	0.193849	0.039564	0	0	0	0	-9.14334357e-05	8.01341277e-02	-1.76908670e+01	0.70
N	F	0.193849	0.391642	0	0	0	0	0	0	0	0
N	Y	0.193849	0.419186	0	0	0	0	0	0	0	0
N	W	0.193849	0.550297	0	0	0	0	0	0	0	0

TABLE S13: Fitness function parameters for ε_{ij} where i = N in Mpipi-T Model 3

Amino Acid i	Amino Acid j	$\varepsilon_{ii,\text{Mpipi}}$	$\varepsilon_{jj,\text{Mpipi}}$	a_i	b_i	c_i	α_i	a_j	b_j	c_j	α_j
P	R	0.078677	0.089916	0	0	0	0	0	0	0	0
P	H	0.078677	0.027216	0	0	0	0	0	0	0	0
P	K	0.078677	0.019117	0	0	0	0	0	0	0	0
P	D	0.078677	0.079096	0	0	0	0	0	0	0	0
P	E	0.078677	0.085622	0	0	0	0	0	0	0	0
P	S	0.078677	0.061600	0	0	0	0	0	0	0	0
P	T	0.078677	0.030758	0	0	0	0	0	0	0	0
P	N	0.078677	0.193849	0	0	0	0	0	0	0	0
P	Q	0.078677	0.200448	0	0	0	0	0	0	0	0
P	C	0.078677	0.069311	0	0	0	0	0	0	0	0
P	G	0.078677	0.096470	0	0	0	0	0	0	0	0
P	P	0.078677	0.078677	0	0	0	0	0	0	0	0
P	A	0.078677	0.049480	0	0	0	0	-6.84807201e-05	5.42590026e-02	-8.46961697e+00	0.70
P	V	0.078677	0.005578	0	0	0	0	-1.49763237e-04	1.15406051e-01	-1.92322375e+01	0.70
P	I	0.078677	0.000395	0	0	0	0	-9.88175293e-05	1.10797972e-01	-1.85218569e+01	0.70
P	L	0.078677	0.010998	0	0	0	0	-1.06970639e-04	1.17281979e-01	-1.91871215e+01	0.70
P	M	0.078677	0.039564	0	0	0	0	-9.14334357e-05	8.01341277e-02	-1.76908670e+01	0.70
P	F	0.078677	0.391642	0	0	0	0	0	0	0	0
P	Y	0.078677	0.419186	0	0	0	0	0	0	0	0
P	W	0.078677	0.550297	0	0	0	0	0	0	0	0

TABLE S14: Fitness function parameters for ε_{ij} where i = P in Mpipi-T Model 3

Amino Acid i	Amino Acid j	$\varepsilon_{ii,\text{Mpipi}}$	$\varepsilon_{jj,\text{Mpipi}}$	a_i	b_i	c_i	α_i	a_j	b_j	c_j	α_j
Q	R	0.200448	0.089916	0	0	0	0	0	0	0	0
Q	H	0.200448	0.027216	0	0	0	0	0	0	0	0
Q	K	0.200448	0.019117	0	0	0	0	0	0	0	0
Q	D	0.200448	0.079096	0	0	0	0	0	0	0	0
Q	E	0.200448	0.085622	0	0	0	0	0	0	0	0
Q	S	0.200448	0.061600	0	0	0	0	0	0	0	0
Q	T	0.200448	0.030758	0	0	0	0	0	0	0	0
Q	N	0.200448	0.193849	0	0	0	0	0	0	0	0
Q	Q	0.200448	0.200448	0	0	0	0	0	0	0	0
Q	C	0.200448	0.069311	0	0	0	0	0	0	0	0
Q	G	0.200448	0.096470	0	0	0	0	0	0	0	0
Q	P	0.200448	0.078677	0	0	0	0	0	0	0	0
Q	A	0.200448	0.049480	0	0	0	0	-6.84807201e-05	5.42590026e-02	-8.46961697e+00	0.70
Q	V	0.200448	0.005578	0	0	0	0	-1.49763237e-04	1.15406051e-01	-1.92322375e+01	0.70
Q	I	0.200448	0.000395	0	0	0	0	-9.88175293e-05	1.10797972e-01	-1.85218569e+01	0.70
Q	L	0.200448	0.010998	0	0	0	0	-1.06970639e-04	1.17281979e-01	-1.91871215e+01	0.70
Q	M	0.200448	0.039564	0	0	0	0	-9.14334357e-05	8.01341277e-02	-1.76908670e+01	0.70
Q	F	0.200448	0.391642	0	0	0	0	0	0	0	0
Q	Y	0.200448	0.419186	0	0	0	0	0	0	0	0
Q	W	0.200448	0.550297	0	0	0	0	0	0	0	0

TABLE S15: Fitness function parameters for ε_{ij} where i = Q in Mpipi-T Model 3

Amino Acid i	Amino Acid j	$\varepsilon_{ii,\text{Mpipi}}$	$\varepsilon_{jj,\text{Mpipi}}$	a_i	b_i	c_i	α_i	a_j	b_j	c_j	α_j
R	R	0.089916	0.089916	0	0	0	0	0	0	0	0
R	H	0.089916	0.027216	0	0	0	0	0	0	0	0
R	K	0.089916	0.019117	0	0	0	0	0	0	0	0
R	D	0.089916	0.079096	0	0	0	0	0	0	0	0
R	E	0.089916	0.085622	0	0	0	0	0	0	0	0
R	S	0.089916	0.061600	0	0	0	0	0	0	0	0
R	T	0.089916	0.030758	0	0	0	0	0	0	0	0
R	N	0.089916	0.193849	0	0	0	0	0	0	0	0
R	Q	0.089916	0.200448	0	0	0	0	0	0	0	0
R	C	0.089916	0.069311	0	0	0	0	0	0	0	0
R	G	0.089916	0.096470	0	0	0	0	0	0	0	0
R	P	0.089916	0.078677	0	0	0	0	0	0	0	0
R	A	0.089916	0.049480	0	0	0	0	-6.84807201e-05	5.42590026e-02	-8.46961697e+00	0.70
R	V	0.089916	0.005578	0	0	0	0	-1.49763237e-04	1.15406051e-01	-1.92322375e+01	0.70
R	I	0.089916	0.000395	0	0	0	0	-9.88175293e-05	1.10797972e-01	-1.85218569e+01	0.70
R	L	0.089916	0.010998	0	0	0	0	-1.06970639e-04	1.17281979e-01	-1.91871215e+01	0.70
R	M	0.089916	0.039564	0	0	0	0	-9.14334357e-05	8.01341277e-02	-1.76908670e+01	0.70
R	F	0.089916	0.391642	0	0	0	0	0	0	0	0
R	Y	0.089916	0.419186	0	0	0	0	0	0	0	0
R	W	0.089916	0.550297	0	0	0	0	0	0	0	0

TABLE S16: Fitness function parameters for ε_{ij} where i = R in Mpipi-T Model 3

Amino Acid i	Amino Acid j	$\varepsilon_{ii,\text{Mpipi}}$	$\varepsilon_{jj,\text{Mpipi}}$	a_i	b_i	c_i	α_i	a_j	b_j	c_j	α_j
S	R	0.061600	0.089916	0	0	0	0	0	0	0	0
S	H	0.061600	0.027216	0	0	0	0	0	0	0	0
S	K	0.061600	0.019117	0	0	0	0	0	0	0	0
S	D	0.061600	0.079096	0	0	0	0	0	0	0	0
S	E	0.061600	0.085622	0	0	0	0	0	0	0	0
S	S	0.061600	0.061600	0	0	0	0	0	0	0	0
S	T	0.061600	0.030758	0	0	0	0	0	0	0	0
S	N	0.061600	0.193849	0	0	0	0	0	0	0	0
S	Q	0.061600	0.200448	0	0	0	0	0	0	0	0
S	C	0.061600	0.069311	0	0	0	0	0	0	0	0
S	G	0.061600	0.096470	0	0	0	0	0	0	0	0
S	P	0.061600	0.078677	0	0	0	0	0	0	0	0
S	A	0.061600	0.049480	0	0	0	0	-6.84807201e-05	5.42590026e-02	-8.46961697e+00	0.70
S	V	0.061600	0.005578	0	0	0	0	-1.49763237e-04	1.15406051e-01	-1.92322375e+01	0.70
S	I	0.061600	0.000395	0	0	0	0	-9.88175293e-05	1.10797972e-01	-1.85218569e+01	0.70
S	L	0.061600	0.010998	0	0	0	0	-1.06970639e-04	1.17281979e-01	-1.91871215e+01	0.70
S	M	0.061600	0.039564	0	0	0	0	-9.14334357e-05	8.01341277e-02	-1.76908670e+01	0.70
S	F	0.061600	0.391642	0	0	0	0	0	0	0	0
S	Y	0.061600	0.419186	0	0	0	0	0	0	0	0
S	W	0.061600	0.550297	0	0	0	0	0	0	0	0

TABLE S17: Fitness function parameters for ε_{ij} where i = S in Mpipi-T Model 3

Amino Acid i	Amino Acid j	$\varepsilon_{ii,\text{Mpipi}}$	$\varepsilon_{jj,\text{Mpipi}}$	a_i	b_i	c_i	α_i	a_j	b_j	c_j	α_j
T	R	0.030758	0.089916	0	0	0	0	0	0	0	0
T	H	0.030758	0.027216	0	0	0	0	0	0	0	0
T	K	0.030758	0.019117	0	0	0	0	0	0	0	0
T	D	0.030758	0.079096	0	0	0	0	0	0	0	0
T	E	0.030758	0.085622	0	0	0	0	0	0	0	0
T	S	0.030758	0.061600	0	0	0	0	0	0	0	0
T	T	0.030758	0.030758	0	0	0	0	0	0	0	0
T	N	0.030758	0.193849	0	0	0	0	0	0	0	0
T	Q	0.030758	0.200448	0	0	0	0	0	0	0	0
T	C	0.030758	0.069311	0	0	0	0	0	0	0	0
T	G	0.030758	0.096470	0	0	0	0	0	0	0	0
T	P	0.030758	0.078677	0	0	0	0	0	0	0	0
T	A	0.030758	0.049480	0	0	0	0	-6.84807201e-05	5.42590026e-02	-8.46961697e+00	0.70
T	V	0.030758	0.005578	0	0	0	0	-1.49763237e-04	1.15406051e-01	-1.92322375e+01	0.70
T	I	0.030758	0.000395	0	0	0	0	-9.88175293e-05	1.10797972e-01	-1.85218569e+01	0.70
T	L	0.030758	0.010998	0	0	0	0	-1.06970639e-04	1.17281979e-01	-1.91871215e+01	0.70
T	M	0.030758	0.039564	0	0	0	0	-9.14334357e-05	8.01341277e-02	-1.76908670e+01	0.70
T	F	0.030758	0.391642	0	0	0	0	0	0	0	0
T	Y	0.030758	0.419186	0	0	0	0	0	0	0	0
T	W	0.030758	0.550297	0	0	0	0	0	0	0	0

TABLE S18: Fitness function parameters for ε_{ij} where i = T in Mpipi-T Model 3

Amino Acid i	Amino Acid j	$\varepsilon_{ii,\text{Mpipi}}$	$\varepsilon_{jj,\text{Mpipi}}$	a_i	b_i	c_i	α_i	a_j	b_j	c_j	α_j
V	R	0.005578	0.089916	-1.49763237e-04	1.15406051e-01	-1.92322375e+01	0.70	0	0	0	0
V	H	0.005578	0.027216	-1.49763237e-04	1.15406051e-01	-1.92322375e+01	0.70	0	0	0	0
V	K	0.005578	0.019117	-1.49763237e-04	1.15406051e-01	-1.92322375e+01	0.70	0	0	0	0
V	D	0.005578	0.079096	-1.49763237e-04	1.15406051e-01	-1.92322375e+01	0.70	0	0	0	0
V	E	0.005578	0.085622	-1.49763237e-04	1.15406051e-01	-1.92322375e+01	0.70	0	0	0	0
V	S	0.005578	0.061600	-1.49763237e-04	1.15406051e-01	-1.92322375e+01	0.70	0	0	0	0
V	T	0.005578	0.030758	-1.49763237e-04	1.15406051e-01	-1.92322375e+01	0.70	0	0	0	0
V	N	0.005578	0.193849	-1.49763237e-04	1.15406051e-01	-1.92322375e+01	0.70	0	0	0	0
V	Q	0.005578	0.200448	-1.49763237e-04	1.15406051e-01	-1.92322375e+01	0.70	0	0	0	0
V	C	0.005578	0.069311	-1.49763237e-04	1.15406051e-01	-1.92322375e+01	0.70	0	0	0	0
V	G	0.005578	0.096470	-2.69573827e-04	1.15406051e-01	-1.92322375e+01	0.70	0	0	0	0
V	P	0.005578	0.078677	-1.49763237e-04	1.15406051e-01	-1.92322375e+01	0.70	0	0	0	0
V	A	0.005578	0.049480	-1.49763237e-04	1.15406051e-01	-1.92322375e+01	0.70	-6.84807201e-05	5.42590026e-02	-8.46961697e+00	0.70
V	V	0.005578	0.005578	-1.49763237e-04	1.15406051e-01	-1.92322375e+01	0.70	-1.49763237e-04	1.15406051e-01	-1.92322375e+01	0.70
V	I	0.005578	0.000395	-1.49763237e-04	1.15406051e-01	-1.92322375e+01	0.70	-9.88175293e-05	1.10797972e-01	-1.85218569e+01	0.70
V	L	0.005578	0.010998	-1.49763237e-04	1.15406051e-01	-1.92322375e+01	0.70	-1.06970639e-04	1.17281979e-01	-1.91871215e+01	0.70
V	M	0.005578	0.039564	-1.49763237e-04	1.15406051e-01	-1.92322375e+01	0.70	-9.14334357e-05	8.01341277e-02	-1.76908670e+01	0.70
V	F	0.005578	0.391642	-1.49763237e-04	1.15406051e-01	-1.92322375e+01	0.70	0	0	0	0
V	Y	0.005578	0.419186	-1.49763237e-04	1.15406051e-01	-1.92322375e+01	0.70	0	0	0	0
V	W	0.005578	0.550297	-1.49763237e-04	1.15406051e-01	-1.92322375e+01	0.70	0	0	0	0

TABLE S19: Fitness function parameters for ε_{ij} where i = V in Mpipi-T Model 3

Amino Acid i	Amino Acid j	$\varepsilon_{ii,\mathrm{Mpipi}}$	$\varepsilon_{jj,\mathrm{Mpipi}}$	a_i	b_i	c_i	α_i	a_j	b_j	c_j	α_j
W	R	0.550297	0.089916	0	0	0	0	0	0	0	0
W	H	0.550297	0.027216	0	0	0	0	0	0	0	0
W	K	0.550297	0.019117	0	0	0	0	0	0	0	0
W	D	0.550297	0.079096	0	0	0	0	0	0	0	0
W	E	0.550297	0.085622	0	0	0	0	0	0	0	0
W	S	0.550297	0.061600	0	0	0	0	0	0	0	0
W	T	0.550297	0.030758	0	0	0	0	0	0	0	0
W	N	0.550297	0.193849	0	0	0	0	0	0	0	0
W	Q	0.550297	0.200448	0	0	0	0	0	0	0	0
W	C	0.550297	0.069311	0	0	0	0	0	0	0	0
W	G	0.550297	0.096470	0	0	0	0	0	0	0	0
W	P	0.550297	0.078677	0	0	0	0	0	0	0	0
W	A	0.550297	0.049480	0	0	0	0	-6.84807201e-05	5.42590026e-02	-8.46961697e+00	0.70
W	V	0.550297	0.005578	0	0	0	0	-1.49763237e-04	1.15406051e-01	-1.92322375e+01	0.70
W	I	0.550297	0.000395	0	0	0	0	-9.88175293e-05	1.10797972e-01	-1.85218569e+01	0.70
W	L	0.550297	0.010998	0	0	0	0	-1.06970639e-04	1.17281979e-01	-1.91871215e+01	0.70
W	M	0.550297	0.039564	0	0	0	0	-9.14334357e-05	8.01341277e-02	-1.76908670e+01	0.70
W	F	0.550297	0.391642	0	0	0	0	0	0	0	0
W	Y	0.550297	0.419186	0	0	0	0	0	0	0	0
W	W	0.550297	0.550297	0	0	0	0	0	0	0	0

TABLE S20: Fitness function parameters for ε_{ij} where i = W in Mpipi-T Model 3

Amino Acid i	Amino Acid j	$\varepsilon_{ii,\mathrm{Mpipi}}$	$\varepsilon_{jj,\mathrm{Mpipi}}$	a_i	b_i	c_i	α_i	a_j	b_j	c_j	α_j
Y	R	0.419186	0.089916	0	0	0	0	0	0	0	0
Y	H	0.419186	0.027216	0	0	0	0	0	0	0	0
Y	K	0.419186	0.019117	0	0	0	0	0	0	0	0
Y	D	0.419186	0.079096	0	0	0	0	0	0	0	0
Y	E	0.419186	0.085622	0	0	0	0	0	0	0	0
Y	S	0.419186	0.061600	0	0	0	0	0	0	0	0
Y	T	0.419186	0.030758	0	0	0	0	0	0	0	0
Y	N	0.419186	0.193849	0	0	0	0	0	0	0	0
Y	Q	0.419186	0.200448	0	0	0	0	0	0	0	0
Y	C	0.419186	0.069311	0	0	0	0	0	0	0	0
Y	G	0.419186	0.096470	0	0	0	0	0	0	0	0
Y	P	0.419186	0.078677	0	0	0	0	0	0	0	0
Y	A	0.419186	0.049480	0	0	0	0	-6.84807201e-05	5.42590026e-02	-8.46961697e+00	0.70
Y	V	0.419186	0.005578	0	0	0	0	-1.49763237e-04	1.15406051e-01	-1.92322375e+01	0.70
Y	I	0.419186	0.000395	0	0	0	0	-9.88175293e-05	1.10797972e-01	-1.85218569e+01	0.70
Y	L	0.419186	0.010998	0	0	0	0	-1.06970639e-04	1.17281979e-01	-1.91871215e+01	0.70
Y	M	0.419186	0.039564	0	0	0	0	-9.14334357e-05	8.01341277e-02	-1.76908670e+01	0.70
Y	F	0.419186	0.391642	0	0	0	0	0	0	0	0
Y	Y	0.419186	0.419186	0	0	0	0	0	0	0	0
Y	W	0.419186	0.550297	0	0	0	0	0	0	0	0

TABLE S21: Fitness function parameters for ε_{ij} where i = Y in Mpipi-T Model 3

[1] H. S. Ashbaugh and H. W. Hatch, Natively unfolded protein stability as a coil-to-globule transition in charge/hydropathy space, Journal of the American Chemical Society **130**, 9536 (2008).

[2] J. D. Weeks, D. Chandler, and H. C. Andersen, Role of repulsive forces in determining the equilibrium structure of simple liquids, The Journal of Chemical Physics **54**, 5237 (1971).

[3] F. G. Quiroz and A. Chilkoti, Sequence heuristics to encode phase behaviour in intrinsically disordered protein polymers, Nature Materials **14**, 1164 (2015).

[4] J. A. Joseph, A. Reinhardt, A. Aguirre, P. Y. Chew, K. O. Russell, J. R. Espinosa, A. Garaizar, and R. Collepardo-Guevara, Physics-driven coarse-grained model for biomolecular phase separation with near-quantitative accuracy, Nature Computational Science **1**, 732 (2021).

[5] A. Bremer, M. Farag, W. M. Borcherds, I. Peran, E. W. Martin, R. V. Pappu, and T. Mittag, Deciphering how naturally occurring sequence features impact the phase behaviours of disordered prion-like domains, Nature Chemistry **14**, 196 (2022).

[6] J. R. Simon, N. J. Carroll, M. Rubinstein, A. Chilkoti, and G. P. López, Programming molecular self-assembly of intrinsically disordered proteins containing sequences of low complexity, Nature Chemistry **9**, 509 (2017).

[7] C. Garcia-Cabau, A. Bartomeu, G. Tesei, K. C. Cheung, J. Pose-Utrilla, S. Picó, A. Balaceanu, B. Duran-Arqué, M. Fernández-Alfara, J. Martín, *et al.*, Mis-splicing of a neuronal microexon promotes cpeb4 aggregation in asd, Nature **637**, 496 (2025).
